# Ozone

**Published:** 1866-04

**Authors:** 


*Ozone.*---The following are the reliable facts known up to this time respecting ozone:. 1. Ozone in a natural state is always present in the air in minute proportions, viz : one in ten thousand. 2. It is destroyed in large towns, and with special rapidity in crowded, close, and filthy localities. 3. Ozone gives to oxygen properties which enable it to support life. In this respect it acts like heat; its effects are destroyed by great heat. 4. Ozone diffused through air in minute quantities produces, on inhalation, distinct symptoms of acute catarrh. 5. When animals are subjected to ozone in large qaantities, the symptoms produced, at a temperature of 75°, are those of inflammation of the throat and mucous membranes generally, and at last congestive bronchitis, which, in carniverous animals, is often rapidly fatal. 6. When animals are subjected for a long period to ozone in small proportions, the agent acts differently, according to the animal. The carnivora die, after some hours, from disorganization of the blood, but the herbivora will live for weeks, and will suffer from no acute disease. 7. The question whether the presence of ozone in the air can produce actual disease, must be answered cautiously. Science has yet no actual *demonstrative* evidence on the point. But the facts approach to demonstration that catarrh is induced by this agent. 8. During periods of intense heat of weather ozone looses its active power. 9. On dead organic matter undergoing putrefaction, ozone acts rapidly; it entirely deodorizes by breaking up the ammoniacal products of decomposition. At the same time it hastens the organic destruction. 10. There is an opposite condition of the air by which the oxygen is rendered negative in action, as compared with the air when it is charged with ozone. Air can thus be rendered negative by merely subjecting it, over and over again, to animals for respiration. The purification of such air from carbonic acid and other tangible impurities, does not render it capable of supporting healthy life; but ozone restores the power. In a negative condition of air, the purification of the organic matter is greatly modified, and the offensive products are increased. Wounds become unhealthy and heal slowly in such negative air. 11. There is no demonstrative evidence, as yet, that any diseases are actually caused by this negative condition of air; but the inference is fair, that diseases which show a putrefactive tendency, are influenced injuriously by a negative condition of the oxygen of the air. It is also probable that during this state, decomposing organic poisonous matters become more injurious. 12. As ozone is used up in crowded localities, and it is essential that ozone should be constantly supplied, in order to sustain the removal of decomposing substances and their products, no more attention to ventilation and other mechanical measures of a sanitary kind can be fully effective, unless the air introduced be made active by ozone. Fever hospitals and other large buildings in towns should be artificially fed with ozonized air.---*Brit. Medical Journal.*

